# Telerehabilitation to Address the Rehabilitation Gap in Anterior Cruciate Ligament Care: Survey of Physical Therapists/Care Providers

**DOI:** 10.1089/tmr.2023.0022

**Published:** 2024-02-13

**Authors:** Elizabeth C. Gardner, Corey Podbielski, Emma Dunphy

**Affiliations:** ^1^ Department of Orthopedics and Rehabilitation, Yale University School of Medicine, New Haven, Connecticut, USA.; ^2^ Outpatient Physical Therapy, Gaylord Speciality Healthcare, Wallingford, Connecticut, USA.; ^3^ Research Department of Primary Care and Populational Health, Upper Third Floor, UCL Medical School (Royal Free Campus), London, United Kingdom.

**Keywords:** ACL, digital health interventions, physical therapy, rehabilitation, telerehabilitation

## Abstract

**Background:**

While the importance of structured rehabilitation following anterior cruciate ligament reconstruction (ACLR), particularly in the return-to-sport phase, is known, for most patients, supervised physical therapy is often completed before this time point. The recent emergence of telerehabilitation and other digital health interventions has the potential to address this “rehabilitation gap.”

**Methods:**

The study was conducted as a cross-section, online survey collecting qualitative and quantitative data from open and closed questions. Inclusion criteria included local qualified physical therapists or other professionals working with ACLR patients.

**Results:**

Eighty-three percent of respondents experienced a “rehabilitation gap” with their ACLR patients. Few reported currently utilizing apps or websites (9.74%). The majority (41/58) reported experience with telerehabilitation, and 84% felt that there was a role for digital rehabilitation strategies to address the “rehabilitation gap.” The vast majority (94.74%) of participants felt that standard commercial insurance did not permit sufficient rehabilitation.

**Discussion:**

While the majority of our respondents acknowledged the existence of a “rehabilitation gap,” as well as familiarity with and confidence in telerehabilitation, few were using this technique at the time of our survey. This suggests an opportunity for development in this space.

## Introduction

An estimated 150,000 to 390,000 anterior cruciate ligament (ACL) injuries occur each year in the United States.^1–4^ The overall age- and sex-adjusted annual incidence of ACL tears was 68.6 per 100,000 person-years, making it a common orthopedic injury.^5^ Surgical reconstruction is commonly used to treat ACL injury with the goal of returning to sports.^6^ Between 2004 and 2014, surgeries increased 5.7-fold.^7^ The cost is estimated at >3 billion dollars annually, not including the psychosocial costs incurred by patients, their families, and clinical teams involved.^8^ The postoperative rehabilitation process can be lengthy^9^ and includes predominantly evidence-based exercise interventions led by physical therapists through clearly defined stages of care.^10–17^ It takes most patients 9 to 12 months after surgery to pass the return-to-sports criteria.^9,18^ However, it has been demonstrated that two-thirds of patients have not returned to preinjury level of competitive sport 12 months after surgery.^19^

The return-to-sport stage of rehabilitation is crucial for those who intend to participate in sports after anterior cruciate ligament reconstruction (ACLR), particularly as returning to sports too soon risks reinjury.^20–22^ It includes complex decision making around when and how to progress through physical and psychological challenges to facilitate optimum function and minimize the risk of reinjury.^20,23^ It requires appropriate return-to-sport tests that recreate the physical challenges of sports in a controlled way and measure the individual's ability to perform the necessary physical tasks of their sport.^12,20,21^ Delaying return to sports until an individual passes these return-to-sport milestone tests helps prevent reinjury.^3,24,25^ Navigating this without a supervising physical therapist presents challenges, particularly as evidence suggests there is a difference between athletes' perceived and actual readiness to return to sports.^26^

A recent web-based survey of >1000 American physical therapists found that rehabilitation practice varied significantly in terms of the content and duration of supervised care.^23^ Specifically, regarding the length of treatment, supervised physical therapy (PT) ranged from 1 to 3 months (15% of respondents) to 12 months (11% of respondents). Overall, 56% of respondents reported 3 to 5 months or less of supervised PT. Another survey-based study was performed on 304 adults 1–20 years following ACLR.^27^ Patients were seen most commonly once a week for the first 3 months (38%) to once every month (26%) from 6 to 9 months. The exploratory analysis identified that longer durations of supervised rehabilitation are associated with a faster return to sport, greater likelihood of return to previous level of sport, and fewer reported ongoing problems with the knee. Unfortunately, 90% of PT visits are in the first 16 weeks after surgery.^28^

These data suggest that many patients leave supervised care before participating in return to sports rehabilitation or achieving a return to sports.^9^ This demonstrates that for many patients, there is a *rehabilitation gap* between the end of care and completion of rehabilitation, including return to sports. Greenberg et al.^23^ concluded that this gap may contribute to patient confusion and suboptimal outcomes.

Telerehabilitation and other digital health interventions (DHIs) may provide options to address this rehabilitation gap. Telerehabilitation can provide evidence-based information, education, and exercise guidance and is acceptable to patients.^29–31^ Some can facilitate accurate measurement of range of motion.^31,32^ However, the challenges of physical assessment and obtaining objective measures are an important limitation despite innovations in this area. This technology has the potential to improve adherence to rehabilitation by engaging with mechanisms of behavior, including personalized features, such as prompts, goal setting, and exercise logs.^33,34^

Telerehabilitation was comparable to conventional in-person rehabilitation in improving clinical outcomes, following knee replacement.^33^ Some of these interventions are wearable,^35^ app or website-based,^33,34^ and some are machines that may be delivered to patient's homes for the rehabilitation period.^36^ The latter study used a three-dimensional camera to facilitate communication between the physical therapists and the patients.^36^ However, the burden of equipment delivery and maintenance in these models present challenges.

A further challenge exists in managing the potential for digital exclusion if patients have limited knowledge or access to technology. Twenty-three percent of Americans may not have access to home broadband.^37^

Although multiple innovations in this area are available to both patients and physical therapists, this article is unique in its exploration of clinician opinions of the role of telerehabilitation for the rehabilitation gap in the management of ACL patients

Digital rehabilitation methods are variously called digital health, eHealth, or mobile health; the term telerehabilitation is increasingly used to describe digital tools that include video consultation. However, in this article, we use the term telerehabilitation as a catch-all term to describe digital rehabilitation strategies that may or may not include video consultations.

Patients have previously used telerehabilitation for knee injuries with high fidelity and noted improved confidence and motivation with their rehabilitation.^22,33,36^ A recent systematic review found that telerehabilitation following joint replacement was less expensive than in-person therapy, with equivalent outcomes and patient satisfaction.^38^

As the rehabilitation gap appears to be often caused by patients only being allowed (or able to afford) a certain number of PT appointments, using telerehabilitation through the rehabilitation process as an adjunct to face-to-face care may create an opportunity to prolong PT by stretching the allocated appointments over a longer period.

The attitudes of ACLR patients toward the use of telerehabilitation were examined in a 2020 survey.^39^ Reported benefits included resource saving, improved access to care, improved learning, and greater engagement. Concerns included incorrect performance of exercises or unmanaged pain being missed and less access to manual therapy, motivation, and opportunities to ask questions.^39^ The attitude of providers toward this technology after ACLR is unknown.

### Objectives

1. Explore PT experiences of delivering ACL care.
2. Explore opinions of the rehabilitation gap between end of care and recovery/return to sport.
3. To understand PT experiences of and opinions of the role of telerehabilitation in ACL care.

## Methods

### Design

This study is a cross-sectional, online survey collecting qualitative and quantitative data from open and closed questions.

### Ethics

The survey and study plan were reviewed by the Yale School of Medicine Institutional Review Board and was deemed exempt from review.

### Survey development

The survey was developed using the Qualtrics survey platform at Yale University. The authors conducted a similar study on the experiences and opinions of patients who had ACLR with regard to telerehabilitation.^39^ This study was adapted to explore professional opinions, but for consistency, the same language was used in the questions where possible. The closed questionnaire was designed based on the evidence base and the professional experience of the authors. Clinical peers were consulted on the content through e-mail, but few changes were recommended. The layout of the questionnaire was constructed by a Qualtrics expert at Yale University and the study was piloted with four physical therapists and an orthopedic surgeon. A patient group reviewed the survey and was influential in the decision to include the open ended as well as closed data.

There were 26 questions in the survey, some of which had multiple parts (Appendix A1). It ran on four screen pages but was responsive to phone screens also. Ten questions provided demographic characteristics of the population such as age, gender, ethnicity, professional identity, years qualified, number of ACLRs managed, type and location of practice, and whether or not Medicaid is included. There were seven questions that pertained to participants' experiences of managing patients with ACLR and there were nine questions about their experiences and opinions of DHIs. Likert scales were used in three questions. The methodological justification and analysis of these was outlined in the earlier article and will not be repeated in this study.^39^ The questions were not randomized, and adaptive questions were not used. Respondents could go back and change their answers if they wished. Time spent on the survey was recorded per question. Items were weighted equally. No incentives were offered to complete the survey.

No survey questions were mandatory to allow participants the choice not to answer a question, although participants were prompted to complete the question once before clicking through. Survey questions were predominantly multiple choice; for those questions where “other” was offered as an option, it was followed by an open text answer option. The survey concluded with three open, qualitative questions.

### Participants, recruitment, and survey distribution

The inclusion criteria were qualified physical therapists or other profession working with ACLR patients, including orthopedic surgeons. People were contacted by e-mail and given a Participant Information Sheet (Appendix A1) outlining the study protocol and consent information highlighting that the participation was entirely voluntary. No formal sample size calculation was used in this formative study.^40^

Survey distribution was facilitated by providing a unique electronic link to survey online through Yale Qualtrics. It was initially sent on April 23, 2021, to the Orthopedic Manual Therapy Special Interest Group (SIG) of the American Physical Therapy Association. Follow-up e-mails were sent on May 26, 2021 through the Orthopedic SIG. The purpose of the survey was reiterated with each e-mail. E-mails were sent to 22 physical therapists. It was our intention to also distribute the survey through the American Orthopedic Society for Sports Medicine, however, this request was declined. Survey was available online from May 2021 until February 2022. Respondents represented 47 unique American postcodes.

### Data analysis

The results are analyzed according to a complete case analysis method. Data were pseudonymized. The Checklist for Reporting Results of Internet E-Surveys (CHERRIES) was used as a reporting standard^41^ (Appendix A2). Responses were collected through the Yale Qualtrics online software, and they were downloaded as an Excel document. The data were prepared for analysis by author E.D.

Data were analyzed using Stata version 17 (StataCorp LLC, College Station, TX, USA). The primary analysis was conducted with descriptive statistics and findings were described in frequencies and percentages. Inferential statistics were also used to explore any relationships between participant demographics, such as age, gender, or years of practice and answers on multiple choice questions. Categorical data were analyzed using Fisher's Exact or Chi-Squared tests. To compare continuous data with categorical data a Kruskal–Wallis (more than two groups) or Wilcoxon rank-sum (two groups) was used. To compare two variables of continuous data, a Spearman's rank correlation was used.

For further exploration, a binary category of “years of practice” was created by dividing participants into 5 or <5 years and >5 years of practice. Five years was chosen by the investigating authors/clinicians as a clinically relevant benchmark of clinical experience.

The qualitative data were analyzed using a pragmatic thematic analysis.^42^ Team members coded the data independently and then subsequently agreed on themes. The analysis is both inductive and deductive where responses are analyzed in relation to the research question, however, new or unexpected topics are included in the analysis.^43^ The themes that arose from each question were grouped and the more developed and discussed themes are emphasized and weighted accordingly. Quotes from participants are used to illustrate themes and to directly relay the participant opinion.^43^ The analysis was conducted using NVivo 11 (QSR International).

### Data protection

Only pseudonymized personal identifying data were collected. The zip code of respondents was recorded but this was removed before the analysis. Qualtrics automatically generated individual identifiers for each respondent based to ensure unique respondents. Identifiers were not assigned to individual computers through the use of cookies. Data were protected by the Yale University firewall. An anonymized dataset with no identifiers or zip codes was sent to University College London for analysis where researcher E.D. is based.

## Results

All survey responses were collected utilizing the provided electronic link. Of those participants who started the survey, all completed it (Table 1). Unfortunately, we do not have a definitive number of potential participants, as survey distribution was through a third-party (APTA Orthopedic Manual Therapy SIG), who was unable to provide us with that information.

**Table 1.** Response Rates

View rate (Ratio of unique survey visitors/unique site visitors)	1 (70:70)
Participation rate (Ratio of unique survey visitors/unique site visitors)	1 (70:70)
Completion rate (Ratio of users who finished the survey/users who agreed to participate)	1 (70:70)

### Characteristics of the respondents

Greater than 80% of respondents were under 40 years old, with over half in their thirties. The overwhelming majority of participants identified as “White” (64/68 94.12%). Only 1 participant was not a physical therapist (1/68, 1.47%); they were orthopedic surgeons. Participants had a mean of 8.7 years of experience (range 1–40 years). Just under half the participants (33/67 49.25%) had seen <10 ALCRs in the previous 2 years with 58/67 (84.56%) seeing <25. The difference between private (37/68 54.41) and hospital based (27/68 39.71) was moderate with a further four working (5.88%) in both settings. Seventy-five percent of participants (51/68) treated patients with Medicaid (Table 2).

**Table 2.** Characteristics of Participants

Characteristics	Frequency (%)
Age, years	
20–29	23 (33.82)
30–39	35 (51.47)
40–49	5 (7.35)
50–59	3 (4.41)
60–69	2 (2.94)
70+	0
Gender	
Female	37 (54.41)
Transgender	0
Nonbinary	0
Male	31 (45.59)
Race/ethnicity	
American Indian or Alaskan Native	0
Asian	1 (1.47)
Black or African American	1 (1.47)
Hispanic or Latino	2 (2.94)
Native Hawaiian or Other Pacific Islander	0
White	64 (94.12)
Professional qualifications	
Orthopedist with fellowship training in sports medicine	1 (1.47)
General orthopedics	0
Physical therapist-DPT	55 (80.88)
Physical therapist-masters	7 (10.29)
Other (all PTs)	5 (7.35)
Number of ACLRs treated in the last 2 years	
<10	33 (49.25)
10–24	25 (37.31)
25–50	6 (8.96)
>50	3 (4.48)
In what setting do you see patients	
Private practice	37 (54.41)
Hospital based/academic	27 (39.71)
Both	4 (5.88)
Do you treat Medicaid/state insurance	
No	17 (25)
Yes	51 (75)
Years of practice, mean (SD)	8.7 (8.57)

ACLR, anterior cruciate ligament reconstruction; DPT, doctor of physical therapy; PT, physical therapy; SD, standard deviation.

### Opinions of physical therapy after ACLR

Participants felt that PT after ACLR should last longer for athletes (8.87 months, standard deviation [SD] 2.73) than for those who were recreationally active (6.47 months, SD 2.37). The measure of association between Years of Practice and Length of PT for recreationally active patients was *p* = 0.0493 (−0.2593) indicating a significant association whereby participants with more experience thought less rehabilitation was needed. However, the correlation coefficient is low indicating that the negative relationship is not a strong one. For PT after ACLR for athletes, the trend was the same showing participants with fewer Years of Practice thought that more rehabilitation was needed after ACLR, but the relationship was weak and should be interpreted cautiously.

The qualitative responses (Box 1) reflected a concern that length of rehabilitation be determined by individual factors such as “activity the patients wish to return to.” Participants further expressed a wish to make rehabilitation length dependent on criteria-based progressions rather than time, referring to “being able to take the patient through all the appropriate stages” and “pass return to sports testing.” They also referred to limits of insurance coverage: “most insurances stop when the patient is functional but never get to work on cutting or sports-related drills.”

One participant offered approaches to reducing need for therapy later in care pathway if the patient can access their own gym: “later stages of therapy are able to be at a reduced frequency, assuming the patient has access to appropriate strengthening equipment.” They identified that to fully “recover,” recreational active people need an average of 10.67 (4.39 SD) months, whereas athletes needed an average of 13.42 months (5.09 SD). Results also showed that those with fewer years of practice thought more recovery time was needed (*p* = 0.026; 12 [12–18]; 12 [10–12]). Likewise, people with fewer years of practice thought athletes need longer to recover (*p* = 0.0164 [0.3112]).

**Box 1.** Opinions and experiences of anterior cruciate ligament rehabilitation Part 2: Qualitative responses

How long should PT last after ACLR	
Theme 1: Depends on the patient and their goals	“Depending highly on the activity the patients wish to return to” “Continuous education and movement impairment diagnosis needs to be evolving throughout the rehab process, as well as being able to take the patient through all the appropriate stages of recovery (ballistics included).”
Theme 2: Rehabilitation through all phases of recovery	“After they pass return to sports testing” “later stages of therapy are able to be at a reduced frequency, assuming the patient has access to appropriate strengthening equipment”
Theme 3: Most PT stops when the resources run out	“most insurances stop when it is the patient is functional but never get to work on cutting or sports related drills” “Dependent on insurance versus self pay”
How long do you think it takes the average patient to fully recover?	
Theme 1: Depends	“Graft types, patient beliefs, fear avoidance all play a part” “depends on sport and competition level” “Depends on activity level/sport contact or non-contact, comorbidities, psychological readiness”
Theme 2: Guided by evidence	“Grafts take 9+ months to heal, longer to fully heal. The demands on the ACL of an athlete require greater forces & strain than recreationally active” “Competitive athletes go back to contact at 9 months typically but don't feel fully recovered until closer to a year with competition under their belt”
How do you manage your patients during the final stages?	
Theme 1. Someone pays or PT works for free	“pro bono” “We see patients well beyond the allotted visits from insurance and the school agrees to pay in full to ensure the athletes are returning to sport healthy and ready.” “I am cash based and see them through this phase”
Theme 2. Digital communication	“videos on phone’ “Provide them my email for future questions “ “reduce frequency of PT with more time in between sessions with more patient instruction on how to progress exercises in between visits”

The vast majority of 94.74% (54/57) participants felt that standard commercial insurance did not permit sufficient rehabilitation after PT. Whereas, the participants were more evenly split on whether Medicaid provided enough rehab with 21/45 (46.67%) saying yes and 24/45 (53.33%) saying no. When asked about other limiting factors for physiotherapy, Copays, Work/School Schedules, and Insurance were of some concern to most participants, however, the group was less concerned about Transportation (Table 3). Ten users completed the question around “other” factors, but did not elaborate in comments as to what those other factors were. The significant measure of association between people who had managed more ACLRs in the last 2 years and “transport” (*p* = 0.015) is likely by chance.

**Table 3.** Opinions and Experiences of Anterior Cruciate Ligament Rehabilitation Part 1 Quantitative Responses

How long should PT last after ACLR	Mean (SD)
For recreationally active patients (months)	6.47 (2.37)
For athletes (months)	8.87 (2.73)
Measures of association	*p* Value (correlation coefficient)
For recreationally active patients	
Years of practice continuous	0.0493 (−0.2593)
For athletes	*p* Value Median (IQR)
By years of practice (0–5, >5)	0.0082 10 (7–12), 8 (6–10)
Years of practice continuous	*p* Value (correlation coefficient) 0.0028 (−0.386)
How long does it take the average person to fully recover after ACLR	
For recreationally active patients (months)	10.67 (4.39)
For athletes (months)	13.42 (5.05)
Measures of association	
For athletes	Median (IQR)
Years of practice (0–5, >5)	0.026 12 (12–18), 12 (10–12)
Years of practice continuous	*p* Value (correlation coefficient) 0.0164 (0.3112)
Do you think that standard commercial insurance permits sufficient physical therapy after ACLR	
No	54 (94.74)
Yes	3 (5.26)
Do you think that Medicaid/state insurance permits sufficient physical therapy after ACLR	
No	24 (53.33)
Yes	21 (46.67)
Do you think that your patients' access to physical therapy is limited by any of the following:	
Copays	
Doesn't limit at all	2 (3.39)
Limits slightly	11 (18.64)
Sometimes limits	34 (57.63)
Usually limits	10 (16.95)
Always limits	2 (3.39)
Transportation	
Doesn't limit at all	10 (16.95)
Limits slightly	20 (33.90)
Sometimes limits	25 (42.37)
Usually limits	4 (6.78)
Always limits	0
Work/school schedules	
Doesn't limit at all	2 (3.39)
Limits slightly	14 (23.73)
Sometimes limits	30 (50.87)
Usually limits	11 (18.64)
Always limits	2 (3.39)
Limitations of insurance coverage	
Doesn't limit at all	0
Limits slightly	2 (3.39)
Sometimes limits	11 (18.64)
Usually limits	28 (47.46)
Always limits	17 (28.21)
Measures of association	
Transportation, as a limiting factor for access to PT	*p* Value (Frequency/%)
No of ACLRs	0.015 (10 16.95%, 20 33.90%, 25 42.37%, 4 6.78%, 0 0% 0 0%)
Do you experience a “rehabilitation gap” between when you have completed care and when your patient is recovered?	
Yes	49 (83.05)
No	10 (16.95)
How do you manage your patients during the final stages?	
Guide them on how to progress with verbal advice education	43 (22.05)
Guide them on how to progress with written or printed handouts	51 (26.15)
Guide them on how to progress using websites or apps	19 (9.74)
Refer them to a personal trainer or athletic trainer	44 (22.56)
Continue to see them as a self-paying patient	29 (14.87)
Other	9 (4.62)

IQR, interquartile range.

Forty-nine out of 59 participants (83.05%) experienced a “Rehabilitation Gap” between the end of the care they offer and the patients' full recovery. Participants managed those patients in end-stage rehabilitation by various means, guiding them on how to progress with verbal advice and education (43/195), written advice or handouts (51/195), apps and websites (19/195), by referring them on to personal/athletic trainers (44/195), or continuing to see them as self-payors (29/195). The denominator of 195 does not reflect a change in participant number, but rather that participants were allowed to click more than one option so there were 195 responses in total.

A further 9 participants clicked “Other,” which include, “the school pays for ongoing rehab,” “the patient pays for ongoing care with strength and conditioning coach,” “videos on their phone,” “space the appointments out so they do last to the end,” “provide e-mail for questions,” “provide screening at milestones,” although for the latter it is unclear if this is pro bono work.

### Opinions of digital health interventions: quantitative

Experience of having used different DHIs was reported with telerehabilitation (41/58) and apps (19/56) the most commonly used. Experience of trying wearables was strongly associated with male gender (*p* = 0.002).

When asked about their capability with digital heath interventions overall, more than half the users felt “Moderately Capable” (31/58, 53.45%), 20/58 (34.49%) felt “Strongly” or “Expertly capable.” Forty seven out of 58 (81.04%) people were “Not at all” or “Minorly concerned” with the potential leak of sensitive information.

When asked if there was a role for digital rehabilitation in filling the “Rehabilitation Gap” 84.48% (49/58) participants said “Yes.” Most preferred to use it in the late phase 41.67% (30/72) although there was a wide variation in the Likert responses as to how suitable each phase was (Table 4). Participants with more years of practice (0–5 years and >5 years) were marginally less likely (*p* = 0.049) to see telerehabilitation as suitable in the mid stage of care—return to function.

**Table 4.** Opinions and Experiences of Digital Health

Have you used DHIs—such as telerehabilitation, apps, websites or computer software—to help guide patient's rehabilitation after any surgery or injury	*n* (%)
Tele-rehabilitation (with video)	
Yes	41 (70.69)
No	17 (29.31)
Apps	
Yes	19 (33.93)
No	37 (66.07)
Websites	
Yes	31 (56.36)
No	24 (43.64)
Wearable technologies	
Yes	9 (16.67)
No	45 (83.33)
Other	
Yes	1 (5.26)
No	18 (94.74)
Measures of association	
Wearable technologies	*p* Value
Gender	0.002 (45 83.33%, 9 16.67%)
How capable do you feel with DHIs?	
Not capable at all	1 (1.72)
Minimally capable	6 (10.34)
Moderately capable	31 (53.45)
Strongly capable	16 (27.59)
Expert capability	4 (6.90)
How concerned are you about the potential leak of sensitive medical information during your use of telerehabilitation?	
Not at all concerned	28 (48.28)
Minorly concerned	19 (32.76)
Moderately concerned	9 (15.52)
Strongly concerned	2 (3.45)
Very strongly concerned	0
Do you think that there is a role for DHIs in fill the “rehabilitation gap” after ACLR?	
Yes	49 (84.48)
No	9 (15.52)
Considering the goals of each phase of ACLR, is any phase particularly amenable to the use of DHIs?	
Early phase—Impairment based	24 (33.33)
Mid phase—Return to function	18 (25.00)
Late phase—Return to sport	30 (41.67)
Considering the goals of each phase of ACLR, how well suited is telerehabilitation to achieve the specific goals of that phase?	
Early phase—Impairment based	
Not suited	13 (22.41)
Minimally well	20 (34.48)
Moderately well	10 (17.24)
Well suited	11 (18.97)
Very well suited	4 (6.90)
Mid phase—Return to function	
Not suited	10 (17.24)
Minimally well	22 (37.93)
Moderately well	21 (36.21)
Well suited	3 (5.17)
Very well suited	2 (3.45)
Late phase—Return to sport	
Not suited	13 (22.41)
Minimally well	16 (27.59)
Moderately well	15 (25.86)
Well suited	11 (18.97)
Very well suited	3 (5.17)
Measures of association	*p* Value *n* (%)
Mid phase—Return to function	
Years of practice (0–5, >5)	0.049 [10 (17.24%), 22 (37.93%), 21 (36.21%), 3 (5.17%), 2 (3.45%)]

DHI, digital health intervention.

### Views on the acceptability of digital health in ACLR: qualitative data

There were three open-text, qualitative questions included in the survey (Table 5). The first question was “What would you need from a digital health intervention to make it successful for you to use?” Four key themes arose: high-quality technology, what the patient needs at home, usability and acceptability, and a fourth theme reflects that 3 participants refuted the idea that DHIs were acceptable in any instance. Box 2 explores the patient quotes that best illustrated these themes. A significant weight of responses described technological features that a DHI should have from quality two-way images to biometric functions to measure range or quality of movement. Participants also required ease of use for both patients and clinicians. There were clear concerns that patients would have access to the space, equipment, and environment suitable for rehabilitation even within their own home to engage in telerehabilitation. This included WiFi and good connections. Patients who did not agree with digital rehabilitation did not elaborate on why.

**Table 5.** Qualitative Themes

Questions	Theme 1	Theme 2	Theme 3	Theme 4	Theme 5
What would you need from a DHI to make it successful for you to use?	High quality technology	What does the patient need at home	Usability and acceptability	DHI's are not acceptable to all	
What, if any, are your primary concerns regarding the use of DHIs following ACLR surgery	Clinical risk	Loss of therapeutic relationship	Quality of and access to technology		
What, if any, do you see as potential benefits to the use of DHIs following ACLR surgery?	Facilitates longer follow up to limit the rehabilitation gap	Overcomes distance	Affordability	Benefits for patient	Improved tracking and monitoring

**Box 2.** What would you need from a digital health intervention to make it successful for you to use? (RPE—rate of perceived exertion)

Theme 1. High-quality technology “Video capabilities, patient feedback section, rpe^*^, biometrics if possible” “Full body videoing on both ends to properly cue and see movements” “Interactive software” “Motion capture analysis” “Safe/secure, clear picture/sound, ability to share video/images”
Theme 2. What does the patient need at home? “Patient access to gym space/equipment” “Confidence in security, accessibility for patients” “Space and access to equipment for the patient.” “Patient home set up, internet connection, good video connection “ “The patient being able to have appropriate setup for full view of functional mobility and assessment” “Reliable high-speed internet, good quality picture”
Theme 3: Technology must have high usability and accessibility “Easily accessible for both patient and provider” “Something simple to use. I have only used telehealth a few times, so it was just a hassle to get logged in and learn the program.” “Easy to use” “Improved user friendliness”
Theme 4: DHI is not a high-quality treatment approach “Minimally efficient for post-op patients” “I don't agree with telehealth, I think the patient is at a disservice” “Better to have the patient in person”

When asked what their primary concerns were about using DHI, the resounding concern was for clinical safety. In Box 3, participant quotes demonstrate concern that important clinical issues such as wound care, swelling, normal range of motion, or proper movement patterns may not be accurately assessed through DHI. Participants also lamented a perceived loss of the therapeutic relationship and in particular, the potential of touch to guide and reassure movement, the ability of communication to reassure, motivate, and engage. The third theme of concern was focused on the quality of the technology and the suitability of environment in which the patient will use it. Hardware and connectivity as well as rehabilitation equipment were discussed.

Participants strongly emphasized the potential of DHI to go some way toward bridging the rehabilitation gap (Box 4). There was also a strong emphasis on the ability of DHI to manage patients over a large geographical area. The experiences of pandemic were referenced by multiple participants as having created distance and the potential of DHI to overcome this. Some participants discussed DHI in terms of having cost-saving potential, although they did not clearly elaborate on how. It was thought that patients themselves might be empowered by conducting their rehabilitation at their own home and therefore somewhat on their own terms. Finally, participants saw the potential of DHI to track and monitor outcome measures in a way that would be clinically very useful.

**Box 3.** What, if any, are your primary concerns regarding the use of digital health interventions following anterior cruciate ligament reconstruction surgery

Theme 1. Clinical risk
“Wound issues in early phase; not observing risky patient behaviors” “Inability to assess fully: palpation of tissues, joint mobility presence/absence; effective view of patient during functional mobility assessment and limitation of manual cuing techniques for motor activation and form during interventions” “Missing impairments like terminal extension or not being able to see subtle movement impairments, no MMT testing.” “Difficulty evaluating swelling and range details”
Theme 2. Loss of therapeutic relationship
“Decreased communication between pt (patient^*^) and PT” “No way to cue hands on or demonstrate in person” “Lots to explain, lots of hands-on cueing and manual in the beginning” “So much hands on is needed in early stages and so much proper cueing/loading in later phases” “Lack of manual intervention for scar mobilization and for addressing fear based guarding for ROM” “Lack of pt buy-in if they aren't coming to sessions in person”
Theme 3: Quality of and access to technology and resources for exercise
“Not being able to really analyze patients' movements from different angles/views “ “Hardware and internet connectivity limitations” “Some folks may lack access to wifi/technology needed” “Limited access to equipment if focus is on home exercise program”

**Box 4.** What, if any, do you see as potential benefits to the use of digital health interventions following anterior cruciate ligament reconstruction surgery

Theme 1. Facilitates longer follow up to limit the rehabilitation gap “Intermittent checks ins during end stage of care. Ability to progress over longer durations. “ “Increased length of time to check in with patients and maintain some level of assistance” “Bridging gap before return to play” “I think it'd be very helpful to use in conjunction with in person visits during the early and mid stages of rehab to 1) save visits to more sport specific (if required) activity in later stages and 2) easier for pt to perform less skilled interventions (especially if insurance/transportation gaps are a concern)” “Reducing amount of visits used initially to promote better coverage at the back end”
Theme 2. Overcomes distance “Reach more people in rural locations or when quarantined” “Being able to help more people that are not able to come in person from other areas of your state, region or nationally/internationally” “May help with transportation barrier” “During COVID we used Telehealth to gain access to our patients (especially at the college level) to assist with their rehab process while they were quarantined to homes all over the country.”
Theme 3. Affordability “Cost effective” “May be more cost effective for those who cannot afford personal training or advanced RTS programs.” “Long-term cheap follow-up and easy tool to monitor patients progress”
Theme 4. Benefits for patient “Easier for patient to build into schedule” “Building a dialogue with patients in the comfort of their home in the early stages post op” “Psychosocial emphasis can still be address, patient independence and confidence building“
Theme 5. Improved tracking and monitoring “More 1:1 care. You're able to give patients 1:1 attention and progress/regress more appropriately. Buy in is higher, compliance is higher” “Easy tool to monitor patients progress” “Tracking home/gym workouts”

## Discussion

Over recent decades, despite advances in surgical technique and technology, the time until return to play following ACLR has been lengthened. With greater study of postoperative rehabilitation, acknowledgment of the persistence of functional, psychological, and strength limitations has led many practitioners, both physical therapists and orthopedic surgeons, to often delay their patient's return to unrestricted activity until closer to 9–12 months (or achievement of return to sport criteria, which usually takes a similar time), with the goal of limiting retears and further injury.^9,18^

The purpose of this investigation was to first query physical therapists' experiences in treating ACL postoperative patients, to explore if a “rehabilitation gap” exists and how clinicians experience it and finally to understand clinician attitudes toward alternatives, such as telerehabilitation.

A review of the demographics of our responding providers, revealed that 58/68 (85.29%) were younger than 40 years of age. This is worth noting, since our population largely trained during the era of greater appreciation for return to play criteria. As such, much our population may not have practiced in the time when return to play at 6 months was the norm.

With regard to their recent professional experience with ACLR patients, 58/68 (86.56%) of our respondents had treated <25 ACLR patients in the last 2 years. This suggests that our physical therapist population likely represents general rather than a specialist group. This would confer greater generalizability to the broader PT provider population.

Our respondents felt that it takes a recreational athlete less time (10.67 months, SD 4.39) to fully recover from ACLR, than competitive athletes (13.42 months, SD 5.05). As these two populations are specifically differentiated based upon the demands of their activities, it is logical that competitive athletes would take longer to achieve full recovery. Current literature suggests that return to play criteria are typically not met until 9–12 months,^9,20,44^ and return to sports before 9 months increases the risk of reinjury by 51%.^21^ One respondent suggested, especially for competitive athlete that there is ongoing progress, perhaps necessitated by the environment of unrestricted play, which occurs after a postoperative athlete is allowed to return to sport: “Competitive athletes go back contact at 9 months typically but don't feel fully recovered until closer to a year with competition under their belt.”

Qualitative responses regarding time to full recovery emphasized two primary themes. The first, that this time is in part defined by the goals of the patient, is seen in the difference between our two athlete populations. The second, that the duration of complete recovery is in part guided by evidence, suggests an appreciation that not all aspects of “healing” or “recovery” can be readily physically appreciated. For example, ongoing research into the process of ligamentization,^45^ neuromuscular retraining,^46^ and psychological readiness^47^ has likely contributed to this appreciation. There are researchers who have suggested that return to sports should be delayed for up to 2 years due to ongoing biological and functional considerations.^28^

For both recreational athletes and competitive athletes, there was a significant effect of years in practice with the opined time until full recovery. Specifically, physical therapists with 5 or less years of continuous practice responded that longer time to recovery was necessary. It should be noted that longer duration of practice does not necessarily equate with more experience treating ACL postoperative patients. The reason for this effect is unclear. It could be postulated that a generally less-experienced physical therapist may be more cautious in risk tolerance. Alternatively, physical therapist with a longer duration of practice may have practiced during the time when it was accepted that patients were “recovered” around 6 months after surgery.

Our respondents' perspective regarding the ideal duration of supervised PT following ACLR follows a similar pattern as above. Specifically, they felt that recreational athletes required on average 6.47 months of PT, whereas competitive athletes needed 8.87 months. A 2018 survey of American Physical Therapy Association members reported that the majority of their respondents treated their postoperative ACL patients for 4–5 months (40.6%) and 6–8 months (32.1%).^48^

As with return to sport timing, physical therapists with 5 or fewer years of continuous professional practice felt that patients in both groups needed a longer course of supervised PT treatment. This same effect was seen in the aforementioned survey of American Physical Therapy Association members.^48^ The authors have theorized that perhaps physical therapists with longer treatment experience feel that they are better able to impart their knowledge, or that more experienced practitioners may have a more established network of community-based alternative providers (such as personal trainers/coaches or athletic trainers). Interestingly in their study, high-volume practitioners and certified specialists also advocated for a longer duration of supervised care. This association with volume was not seen in our population.

Prior study of the actual duration of PT following ACLR has been sparse. Two large database studies, both utilizing the PearlDiver database, have been reported.^49,50^ Both have reported an average of 17 postoperative PT sessions per patient. Patients completed a mean of 52% of their visits in the first 6 weeks, 75% in the first 10 weeks, and 90% in the first 16 weeks after surgery.^49^

Prior investigation has suggested that from the perspective of the patient, there may be many reasons for the cessation of formal PT, including the financial cost to and time constraints of the patient and insurance coverage.^23,39^

Themes expressed regarding the optimal duration of supervised PT were similar to those about overall patient recovery with one exception – the impact of “resources” on the duration of treatment, specifically, the impact of insurance coverage on the ability of patients to access PT. This effect was further investigated with direct questions on the topic. Nearly all respondents, 54/57 (94.74%) who answered the question, felt that standard commercial insurance did not permit sufficient PT after ACLR.

It should be noted that in a prior study, 77% of patients felt that they had sufficient PT after ACLR, despite only 11% of patients feeling that they had fully recovered at the end of their PT sessions.^39^

Interestingly, our study population was more split regarding adequacy of PT for Medicaid/state insurance patients, with nearly half of respondents feeling that this population does receive sufficient access to PT. There has been no formalized study of the access of patients to PT based upon their insurance coverage, which limits interpretation of this finding. Additionally, Medicaid visit restrictions vary by state and diagnosis, making it difficult to generalize our findings, as all respondents to our survey were located in a single state.

### Rehabilitation gap

However, despite known ongoing physical deficits, most patients are discharged from supervised PT well before they are cleared for return to unrestricted physical activity, creating what has been referred by these authors as a “rehabilitation gap.”^39^ While other rehabilitation professionals, such as school athletic trainers, may help to assume treatment during this time, a 2015 study reported that only 37% of public secondary schools provide full-time athletic training services,^51^ never mind the population of older patients who are no longer in secondary school.

When asked directly, 83.05% of our respondents felt that there is a “rehabilitation gap” present between the completion of their supervised care and the full recovery of their patient. The most popular strategies to manage this were verbal advice/education, written/printed handouts, and referral to a personal or athletic trainer. While 84.48% of our respondents felt that there is a role for DHIs to fill the “rehabilitation gap,” only 9.74% reported the use of websites or apps, suggesting an opportunity for development in this space.

In the post-COVID19 era, when medical practitioners of all types, including physical therapists, were asked to make rapid adjustments to their practice styles, facilitating the adoption of virtual treatment strategies such as telehealth and websites. In our population, 70.69% of respondents had utilized telerehabilitation with video and 56.36% have referred to websites. Only 33.93% have utilized apps, perhaps suggestive of the lack of developed app resources. Study of both patients and physical therapists have found both groups to be receptive to virtual therapy sessions.^52^ Separate studies have reported the acceptability^29^ and feasibility^33^ of a web-based digital health tool to complement traditional in-person treatment. Our study reported that the majority of practitioners had only minor concerns regarding the potential for leak of sensitive medical information.

The majority of respondents stated that the late phase of rehabilitation, specifically around return to play, would be particularly amenable to the use of DHIs. This is in contradistinction to the opinion of many patients—in prior research by these authors, 60% of survey postoperative patients expressed that they would prefer the use of telerehabilitation early in treatment, to save in-person visits for later.^39^ Many patients did note the potential for telerehabilitation as a maintenance tool following discharge from PT. It is important to note that these patient surveys were collected before the COVID-19 pandemic.

### Limitations

Like all survey studies, our results are limited to the data gathered. Ours had a relatively small sample size, which was limited to a single state, which limits the generalizability to the rendered opinions. One respondent was a sports medicine-trained orthopedic surgeon. At the time of study inception, it was intended that this survey would be distributed to a population of both physical therapists treating postoperative ACL patients, as well as orthopedic surgeons who perform ACLR. However, potentially due to a sudden increase in survey studies during the COVID19 pandemic, distribution of this study through several orthopedic societies was declined. Therefore, the respondent population was limited to physical therapists. The opinion of this important stakeholder population would greatly add to this conversation.

## Conclusions

In an attempt to limit the risk of ACL reinjury, the duration of the return to recreational and competitive sport phase of rehabilitation has lengthened postoperatively. However, the duration of supervised PT has not increased, suggesting that many patients are discharged before full recovery, creating a “rehabilitation gap.” There is great variation in current management of this time period, and DHIs have been identified as one possible resource. Further investment in the development of evidence-based virtual or web-based rehabilitation resources could be a key tool for practitioners and patients alike.

## Abbreviations Used

ACL: anterior cruciate ligament
ALCR: anterior cruciate ligament reconstruction
DHI: digital health intervention
IQR: interquartile range
PT: physical therapy
SD: standard deviation
SIG: Special Interest Group

## Appendix

### Appendix A1. **Dear Anterior Cruciate Ligament Medical Professionals**

We hope that you are doing well and thank you for considering participating in our survey. The purpose of this survey is to explore the opinions and acceptability of the integration of telerehabilitation into the postsurgical treatment of anterior cruciate ligament (ACL) patients. We estimate that this survey will take ∼5 min to complete.

The primary risk of this study is to the confidentiality of your data, which is collected anonymously. To protect your data, all information will be stored on a password-protected computer accessible only to approved research personnel. While you will experience no personal benefit from participating in this research study, knowledge gained may allow us to expand our treatment options for our patients following ACL surgery.

Your decision to participate in the research is completely voluntary. Please reach out to one of our researchers, Corey Podbielski, PT, DPT, OCS (cpodbielski@gaylord.org) if you have any questions or concerns.

If you have questions about your rights as a research participant, or you have complaints about this research, you call the Yale Institutional Review Boards at (203) 785-4688 or e-mail hrpp@yale.edu.

Below you will be asked whether you consent to proceed with the study. If you do not consent, please exit this survey.

Thank you for your time and consideration.

Corey Podbielski, PT, DPT, OCS

Gaylord Physical Therapy

Elizabeth Gardner, MD, FAAOS

Department of Orthopedic Surgery Yale University School of Medicine

 I consent to participate

 I do not consent to participate

Basic Information

Please select your age bracket.

 20–29 years old

 30–39 years old

 40–49 years old

 50–59 years old

 60–69 years old

 70+ years old

What is your gender identity?

 Female

 Transgender

 Nonbinary Male

What is your race/ethnicity?

 American Indian or Alaska Native

 Asian

 Black or African American

 Hispanic or Latino

 Native Hawaiian or Other Pacific Islander White

Please clarify your professional qualifications.

 General Orthopedics

 Orthopedist with fellowship training in Sports Medicine

 Physical therapist—DPT

 Physical therapist—Masters Other

How many years have you been in practice?

Years

How many ACL reconstructions have you performed/rehabilitated in the last 2 years?

 Less than 10

 10–24 times

 25–50 times

 More than 50

In what setting do you see ACL patients?

 Private practice

 Hospital based/academic, Both

Do you treat patients with Medicaid/state insurance?

 Yes

 No

Please provide the zip code of the location in which you provide the majority of your care.

How long do you think that physical therapy should last after ACL reconstruction surgery for the average patient? Please comment if helpful.

For recreationally active patients (number of months), for competitive athlete patients (number of months)

Comments:

How long do you think it takes the average patient to fully “recover” after ACL reconstruction surgery?

For recreationally active patients

(number of months)

For competitive athlete patients

(number of months) Comments

On average, do you think that standard commercial insurance permits sufficient physical therapy after ACL reconstruction surgery?

 Yes

 No

 I Don't Know

On average, do you think that Medicaid/state insurance permits sufficient physical therapy after ACL reconstruction surgery?

 Yes

 No

 I Don't Know

How much do you think that your patients' access to physical therapy is limited by any of the following:

**Table:**

	1 -Doesn't limit at All	2 Limits Slightly	3 - Sometimes limits	4 - Usually limits	5 - Always limits	Click to write Scale Point 6
Copays						
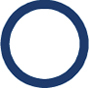
Transportation						
Work/School Schedules						
Limitations of Insurance						
Coverage						

Other

Do you experience a “Rehabilitation Gap” in your practice between when you have completed care and when your patient is fully recovered?

 Yes

 No

Evidence suggests that most patients have completed face-to-face physical therapy 5 months after surgery. How do you manage your patients during the final stages? (Select as many as apply)

 Guide them on how to progress with verbal advice and education

 Guide them on how to progress with written or printed handouts

 Guide them on how to progress using websites or apps

 Refer them to a personal trainer or athletic trainer

 Continue to see them as a self-paying patient, Other

 Have you used digital health interventions, such as tele-rehabilitation, apps, websites, or computer software, to help guide patient's rehabilitation after any surgery or injury?

Yes/No

Tele-rehabilitation (with video)

Apps

Websites

Wearable Technologies

Other

How capable do you feel with digital health interventions?

**Table:**

1 - Not capable at,	2 – Minimally capable,	3 – Moderately capable,	4 - Strongly capable,	5 - Expert all capability
				

How concerned are you about the potential leak of sensitive medical information during your use of tele-rehabilitation?

**Table:**

1 - Not at all concerned,	2 – Minorly concerned,	3 – Moderately concerned,	4 – Strongly concerned,	5 - Very strongly concerned
				

Do you think that there is a role for digital health interventions to fill the “Rehabilitation Gap” after ACL reconstruction?

 Yes

 No

Considering the goals of each phase of ACL rehabilitation, is any phase particularly amenable to the use of digital health interventions? (Choose as many as apply)

 Early Phase - Impairment Based

 Mid Phase - Return to Function

 Late Phase - Return to Sport

Considering the goals of each phase of ACL rehabilitation, how well suited is telerehabilitation (with video capability) to achieve the specific goals of that phase?

**Table:**

	1 - Not suited at all,	2 - Minimally well suited,	3 -Moderately well suited,	4 - Well suited,	5 - Very well suited
Early Phase - Impairment Based					
Mid Phase - Return to Function					
Late Phase - Return to Sport					

For the following 3 questions, we want to hear your thoughts on digital health interventions.

What would you need from a digital health intervention to make it successful for you to use?

What, if any, are your primary concerns regarding the use of digital health interventions following ACL reconstruction surgery?

What, if any, do you see as potential benefits to the use of digital health interventions following ACL reconstruction surgery?

Powered by Qualtrics

### Appendix A2. Checklist for Reporting Results of Internet E-Surveys (CHERRIES)

**Checklist for Reporting Results of Internet E-Surveys (CHERRIES)**

**Table:**

Item category	Checklist item	Explanation
**Design**	Describe survey design	Describe target population, sample frame. Is the sample a convenience sample? (In “open” surveys this is most likely.)
**IRB (Institutional Review Board) approval and informed consent process**	IRB approval	Mention whether the study has been approved by an IRB.
	Informed consent	Describe the informed consent process. Where were the participants told the length of time of the survey, which data were stored and where and for how long, who the investigator was, and the purpose of the study?
	Data protection	If any personal information was collected or stored, describe what mechanisms were used to protect unauthorized access.
**Development and pretesting**	Development and testing	State how the survey was developed, including whether the usability and technical functionality of the electronic questionnaire had been tested before fielding the questionnaire.
**Recruitment process and description of the sample having access to the questionnaire**	Open survey vs. closed survey	An “open survey” is a survey open for each visitor of a site, while a closed survey is only open to a sample, which the investigator knows (password-protected survey).
	Contact mode	Indicate whether or not the initial contact with the potential participants was made on the Internet. (Investigators may also send out questionnaires by mail and allow for Web-based data entry.)
	Advertising the survey	How/where was the survey announced or advertised? Some examples are offline media (newspapers), or online (mailing lists – If yes, which ones?), or banner ads (Where were these banner ads posted and what did they look like?). It is important to know the wording of the announcement as it will heavily influence who chooses to participate. Ideally, the survey announcement should be published as an appendix.
**Survey administration**	Web/E-mail	State the type of e-survey (e.g., one posted on a website, or one sent out through e-mail). If it is an e-mail survey, were the responses entered manually into a database, or was there an automatic method for capturing responses?
	Context	Describe the website (for mailing list/newsgroup) in which the survey was posted. What is the website about, who is visiting it, what are visitors normally looking for? Discuss to what degree the content of the website could preselect the sample or influence the results. For example, a survey about vaccination on a anti-immunization website will have different results from a Web survey conducted on a government website
	Mandatory/voluntary	Was it a mandatory survey to be filled in by every visitor who wanted to enter the website, or was it a voluntary survey?
	Incentives	Were any incentives offered (e.g., monetary, prizes, or nonmonetary incentives such as an offer to provide the survey results)?
	Time/Date	In what timeframe were the data collected?
	Randomization of items or questionnaires	To prevent biases items can be randomized or alternated.
	Adaptive questioning	Use adaptive questioning (certain items, or only conditionally displayed based on responses to other items) to reduce number and complexity of the questions.
	Number of Items	What was the number of questionnaire items per page? The number of items is an important factor for the completion rate.
	Number of screens (pages)	Over how many pages was the questionnaire distributed? The number of items is an important factor for the completion rate.
	Completeness check	It is technically possible to do consistency or completeness checks before the questionnaire is submitted. Was this done, and if “yes,” how (usually JAVAScript)? An alternative is to check for completeness after the questionnaire has been submitted (and highlight mandatory items). If this has been done, it should be reported. All items should provide a nonresponse option such as “not applicable” or “rather not say,” and selection of one response option should be enforced.
	Review step	State whether respondents were able to review and change their answers (e.g., through a Back button or a Review step, which displays a summary of the responses and asks the respondents if they are correct).
**Response rates**	Unique site visitor	If you provide view rates or participation rates, you need to define how you determined a unique visitor. There are different techniques available, based on IP addresses or cookies or both.
	View rate (Ratio of unique survey visitors/unique site visitors)	Requires counting unique visitors to the first page of the survey, divided by the number of unique site visitors (not page views!). It is not unusual to have view rates of <0.1% if the survey is voluntary.
	Participation rate (Ratio of unique visitors who agreed to participate/unique first survey page visitors)	Count the unique number of people who filled in the first survey page (or agreed to participate, e.g., by checking a checkbox), divided by visitors who visit the first page of the survey (or the informed consents page, if present). This can also be called “recruitment” rate.
	Completion rate (Ratio of users who finished the survey/users who agreed to participate)	The number of people submitting the last questionnaire page, divided by the number of people who agreed to participate (or submitted the first survey page). This is only relevant if there is a separate “informed consent” page or if the survey goes over several pages. This is a measure for attrition. Note that “completion” can involve leaving questionnaire items blank. This is not a measure for how completely questionnaires were filled in. (If you need a measure for this, use the word “completeness rate.”)
**Preventing multiple entries from the same individual**	Cookies used	Indicate whether cookies were used to assign a unique user identifier to each client computer. If so, mention the page on which the cookie was set and read, and how long the cookie was valid. Were duplicate entries avoided by preventing users' access to the survey twice; or were duplicate database entries having the same user ID eliminated before analysis? In the latter case, which entries were kept for analysis (e.g., the first entry or the most recent)?
	IP check	Indicate whether the IP address of the client computer was used to identify potential duplicate entries from the same user. If so, mention the period of time for which no two entries from the same IP address were allowed (e.g., 24 h). Were duplicate entries avoided by preventing users with the same IP address access to the survey twice; or were duplicate database entries having the same IP address within a given period of time eliminated before analysis? If the latter, which entries were kept for analysis (e.g., the first entry or the most recent)?
	Log file analysis	Indicate whether other techniques to analyze the log file for identification of multiple entries were used. If so, please describe.
	Registration	In “closed” (non-open) surveys, users need to login first and it is easier to prevent duplicate entries from the same user. Describe how this was done. For example, was the survey never displayed a second time once the user had filled it in, or was the username stored together with the survey results and later eliminated? If the latter, which entries were kept for analysis (e.g., the first entry or the most recent)?
**Analysis**	Handling of incomplete questionnaires	Were only completed questionnaires analyzed? Were questionnaires, which were terminated early (where, e.g., users did not go through all questionnaire pages), also analyzed?
	Questionnaires submitted with an atypical timestamp	Some investigators may measure the time people needed to fill in a questionnaire and exclude questionnaires that were submitted too soon. Specify the timeframe that was used as a cutoff point, and describe how this point was determined.
	Statistical correction	Indicate whether any methods, such as weighting of items or propensity scores, have been used to adjust for the nonrepresentative sample; if so, please describe the methods.
